# 

**Published:** 1888-10-20

**Authors:** 


PRICE ONE PENNY.

No. 108, Vol. V.—October 20th, 1888.

[Registered at the General Post Office as a Newspaper.]

Per Annum, 6s. 6d., post free.

Monthly Parts, 6d.; post free, 7½d.

Ditto per Ann., 7s. 6d. post free.

### THE HOSPITAL

A WEEKLY INSTITUTIONAL JOURNAL OF

